# Ecometabolomics reveal physiological adaptations of Asiatic toads (*Bufo gargarizans* Cantor, 1842) to different environments along an altitudinal gradient

**DOI:** 10.1186/s12983-025-00577-z

**Published:** 2025-08-15

**Authors:** Mingxue Jiao, Yanghuilin Zhang, Cheng Liu, Zhonghong Cao, Kenneth B. Storey, Yonggang Niu

**Affiliations:** 1 Department of Life Sciences, Dezhou University, Dezhou, 253023 Shandong China; 2 School of Life Sciences and Medicine, Shandong University of Technology, Zibo, China; 3 Department of Biology, Carleton University, Ottawa, ON K1S 5B6 Canada

**Keywords:** *Bufo gargarizans*, Altitudinal gradient, Metabolomics, Adaptation

## Abstract

Animals inhabiting altitudinal gradients exhibit a variety of adaptations to environmental variations. However, to date, changes in metabolomic profiles with altitude have been poorly characterized. Here, we used target and non-target metabolomic analyses of liver to investigate the environmental adaptations of Asiatic toads (*Bufo gargarizans*) along an altitudinal gradient (50 m, 1200 m, 2300 m, and 3400 m above sea level). Non-targeted metabolomics analysis identified 775 metabolites, and k-means clustering analysis showed that up-regulated metabolites along the altitudinal gradient were significantly enriched in the thiamine and sphingolipid metabolism pathways. Down-regulated metabolites were mainly enriched in alanine, aspartate and glutamate metabolism and glycolysis/glycogenesis. Weighted gene co-expression network analysis showed that metabolites positively correlated with altitude were mainly related to sphingolipid metabolism and glycerophospholipid metabolism, whereas those negatively correlated were involved in glycolysis/gluconeogenesis and glycerolipid metabolism. Moreover, a total of 52 metabolites were identified by the targeted metabolomics analysis. K-means clustering analysis showed that down-regulated metabolites along the altitudinal gradient were mainly enriched in pentose phosphate pathway and glycolysis/gluconeogenesis. In addition, toads from different altitudes exhibited significant variation in the activities of key metabolic enzymes, including phosphofructokinase, lactate dehydrogenase, and* α*-ketoglutarate dehydrogenase. In conclusion, the metabolic profiles of Asiatic toads along an altitudinal gradient differed significantly. These findings enhance our understanding of the physiological adaptations of toads to different environments along an altitudinal gradient.

## Background

Altitudinal gradients drive variation in environmental factors such as atmospheric pressure, partial pressure of oxygen, temperature, precipitation, and ultraviolet radiation (UVR), providing a valuable study system for investigating the evolution and adaptation of organisms to geographic conditions [1, 2]. It has been reported that UVR increases by 9% for each 1000 m of altitude and that mean annual surface air temperature decreases with rising altitude at a lapse rate of − 3.4 °C km^−1^ [3, 4]. Moreover, both atmospheric pressure and inspired oxygen pressure decline roughly linearly with rising altitude and are just 50% of the sea level value at 5500 m above sea level [5]. Along an altitudinal gradient these environmental factors can change significantly, thereby affecting the performance and distribution of animals [6, 7]. Therefore, understanding the potential relationship between physiological performance and altitude may be of great importance in revealing adaptations to extreme environments.

The survival and thriving of animals in different environments, especially in extreme high-altitude conditions, depends on adaptations at morphological, physiological, biochemical, and molecular levels. Numerous studies have confirmed that metabolic adjustments play a crucial role in the adaptation of animals to high-altitude environments and have been extensively assessed in endotherms [8, 9]. For instance, compared to lowland populations, highland deer mice (*Peromyscus maniculatus*) have a higher aerobic capacity, thanks to enhanced ventilation and circulation [10, 11], as well as increased capillary and mitochondrial density in skeletal muscle [12]. Moreover, highland deer mice have a greater thermogenic capacity by enhancing lipid metabolism under hypoxia compared with lowland conspecifics [13]. Different metabolic responses have also been observed in a few reptiles from different altitudes. For example, high-altitude resident red-tailed sand lizards, *Phrynocephalus erythrurus*, exhibit lower mitochondrial respiration rates and lactate dehydrogenase activity and higher* β*-hydroxyacetyl-coenzyme A dehydrogenase activity than their low-altitude relatives, *P. przewalskii* [14]. A recent study found a remodeling of metabolic profiles among high-altitude lizards, *P. vlangalii*, favored the utilization of carbohydrates over lipids and showed reliance on maintaining membrane fluidity and structural integrity (i.e., membrane homeostasis) to cope with low-temperature stress at high altitudes [15]. However, information regarding the metabolic adaptations of amphibians to high altitude is less available. Probing metabolic regulation of high altitude among amphibians is significant for revealing adaptations of ectothermic vertebrates to extreme environments. In addition, increasing evidence has shown that metabolomics is an effective tool for gaining an overall view of the changing patterns of many metabolites in different cellular pathways and for analyzing the interactions between organisms and their environment [16]. Metabolomic analyses can be categorized as either non-targeted or targeted approaches [17]. The former involves the broad identification of metabolites in a biological sample without preference [18], whereas the latter enables precise quantification of a predetermined set of substances [17]. Metabolomics has been broadly applied to characterize the response of organisms to a variety of environmental stressors [16], such as cold and fasting in winter [19, 20], low oxygen levels and low temperatures at high altitude [15], as well as exposure to pesticides and predation [21]. Metabolomic analysis has become favored for elucidating biochemical mechanisms underlying physiological processes. However, utilizing this approach to reveal metabolites and metabolic profiles in response to altitudinal gradients in widely distributed amphibians remains deficient.

The Asiatic toad (*Bufo gargarizans*), a common amphibian species, has a broad altitudinal distribution (ranging from sea level to over 4300 m) in East Asia, extending from lowlands to highlands such as the Qinghai-Tibet Plateau [22]. Therefore, this species presents an ideal model for exploring the adaptations of ectothermic vertebrates to different environments. *B. gargarizans*, a traditional medicinal animal, is well known for the pharmaceutical value of its skin secretions and cortex [23]. Recently, a high-quality genome of the Asiatic toad was sequenced and will provide information for uncovering the genetic basis of many biological processes [24]. Asiatic toads can live at high altitude due to physiological changes, such as increased hemoglobin concentration, hematocrit, and red blood cell count [25]. Histological evidence also suggests that Asiatic toads have downsized their brain volume as an adaptation to the high-altitude environment [26]. Comparative transcriptomics also revealed that the expression of genes related to O_2_ transportation were up-regulated in heart of *B. gargarizans* along an altitudinal gradient (790 m, 1500 m, and 2500 m) [27]. Moreover, genes related to nutrient metabolism were down-regulated to a large extent in liver samples from high-altitude toads (3464 m) as compared to low-altitude individuals (559 m) [28]. After comparing Asiatic toad populations at four altitudes (50 m, 1200 m, 2300 m, and 3400 m), it was found that high-altitude toads had stronger antioxidant defenses and adopted a metabolic inhibition strategy to adapt to the plateau environment [29]. However, the metabolic adaptations, especially the key metabolites, metabolic pathways, and metabolic enzyme activities, that are responsible for the ability to cope with environmental conditions at different altitudes remain unknown.

In the present study, we selected the liver of Asiatic toads, a core organ of energy metabolism, from four altitudes (50 m, 1200 m, 2300 m, and 3400 m) and used multi-platform non-targeted analyses (including UHPLC-QE-MS and GC-TOF–MS) and targeted (HPIC-MS/MS) metabolomics methods to analyze metabolic responses to different environments with altitudinal variations. Moreover, the activity of metabolic enzymes in liver also was assayed, including hexokinase (HK), 6-phosphoglucokinase (G6PDH), phosphofructokinase (PFK), pyruvate kinase (PK), pyruvate dehydrogenase (PDH), lactate dehydrogenase (LDH), isocitrate dehydrogenase (ICDH),* α*-ketoglutarate dehydrogenase (*α*-KGDH), succinate dehydrogenase (SDH), and glutamate dehydrogenase (GDH). Comparative metabolomics not only detects changes in circulating small molecules in the body, but also enables the discovery of metabolic pathways associated with environmental stresses. This study helps to provide new insights into the metabolic mechanisms of ectothermic vertebrates adapting to their environments at different altitudes.

## Methods

### Sample collection

A total of 96 adult male toads were collected from four previously reported sites [29], during July 2024 (Table 1). Species identity of *B. gargarizans* was confirmed using morphological characters described by Fei et al. [22], and these four sampled populations belong to the same evolutionary clade of Asiatic toads according to the biogeographical analysis [30]. Individuals (*n* = 24 at each altitude) were euthanized by destroying spinal cord tissue near the sampling site. Liver samples were then quickly dissected and immediately fast-frozen in liquid nitrogen. After transport to the lab at Dezhou University, liver samples were shipped on dry ice to Biotree Biotech Co., Ltd. (Shanghai, China) for analysis of multi-platform non-targeted metabolomics (UHPLC-QE-MS, GC-TOF–MS) (*n* = 10 at each altitude) and targeted metabolomics (HPIC-MS/MS) (*n* = 6 at each altitude). Moreover, liver samples were assessed for enzyme activity (*n* = 8 at each altitude).

**Table 1:** Morphological parameters of toads and geographic coordinates of the sampling sites in this study

Population	Number	Mean body mass (g)	Mean snout-vent length (cm)	Longitude (°E)	Latitude (°N)	Average annual temperature (℃)	Altitude (m)	Locality
1	24	69.60 ± 4.36 a	9.38 ± 0.32 a	116.78	37.53	13.3	50	Dezhou City, Shandong Province
2	24	62.73 ± 3.67 ac	9.10 ± 0.20 a	105.91	34.55	11.8	1200	Tianshui City, Gansu Province
3	24	44.26 ± 3.04 bc	8.35 ± 0.14 ac	103.97	34.63	6.6	2300	Minxian, Gansu Province
4	24	34.67 ± 0.56 b	7.16 ± 0.10 bc	101.87	33.67	2.3	3400	Maqu County, Gansu Province

The morphological data are expressed as the means ± SEM. Different lower-case letters indicate a significant difference (*P* < 0.05) between different altitudes. Data for average annual temperature is downloaded from the National Meteorological Information Center of China (http://data.cma.cn/)

### Metabolites extraction

Metabolites extracted from liver samples (*n* = 10 for each altitude) were subjected to UHPLC-QE-MS and GC-TOF–MS analysis according to our previous studies [15, 31]. Briefly, 50 mg amounts of liver samples were added into 1 mL of ice-cold acetonitrile:methanol:water (2:2:1 v:v:v, containing L-2-chlorophenylalanine as an internal standard). The mixture was then vortexed for 30 s, homogenized at 35 Hz for 4 min using a tissuelyser (Tissuelyser-24L, Shanghai Jingxin Industrial Development Co., LTD) and sonicated for 5 min in an ice-water bath. Then samples were incubated for 1 h at − 40 °C and then centrifuged at 13,800 g for 15 min at 4 °C. Finally, the resulting supernatants were transferred to fresh glass vials for LC–MS/MS analysis. Quality control (QC) samples were prepared by pooling the equivalent supernatants of all samples.

Subsequently, 50 mg samples of liver were added into 1 mL aliquots of ice-cold acetonitrile:methanol:water (2:2:1 v:v:v, containing 0.5% ribitol). The mixture was then homogenized at 35 Hz for 4 min using the tissuelyser and sonicated for 5 min in an ice-water bath. Then the samples were incubated for 1 h at − 40 °C and centrifuged at 13,800 g for 15 min at 4 °C. The resulting supernatant (200 μL) samples were transferred to 1.5 mL EP tubes. QC samples were prepared by pooling the equivalent supernatants (40 μL) of all samples. These extracts were dried in a vacuum concentrator and incubated at 80 °C for 30 min after 40 μL of methoxyamination hydrochloride (20 mg mL^−1^ in pyridine) was added. The derivatization was performed using 50 μL of BSTFA regent (1% TMCS, v:v) at 70 °C for 1.5 h. After cooling, 5 μL of FAMEs (in chloroform) was added to the QC sample.

### UHPLC-QE-MS non-targeted metabolomics

LC–MS/MS analyses were performed using an UHPLC system (Vanquish, Thermo Fisher Scientific) with a UPLC BEH Amide column (2.1 mm × 100 mm, 1.7 μm) coupled to Q Exactive HFX mass spectrometer (Orbitrap MS, Thermo). Flow rate was 400 μL min^−1^ and the injection volume was 4 μL. The mobile phase consisted of 25 mM ammonium acetate and 25 mM ammonia hydroxide in water (pH = 9.75) (A) and acetonitrile (B). The auto-sampler temperature was 4 °C, and the injection volume was 2 μL. The optimized conditions were as follows: sheath gas flow rate, 30 Arb; aux gas flow rate, 25 Arb; capillary temperature, 350 °C, full MS resolution, 60,000; MS/MS resolution, 7500; collision energy, 10/30/60 in NCE mode; spray voltage, 3.6 kV (positive) or − 3.2 kV (negative), respectively. The raw data were converted to the mzXML format using ProteoWizard and processed with an in-house program, which was developed using R and based on XCMS, for peak detection, extraction, alignment, and integration [15, 31]. Then an in-house MS2 database (BiotreeDB) was applied in metabolite annotation. The cutoff for annotation was set at 0.3.

### GC–MS/MS non-targeted analysis

GC-TOF–MS analysis was performed using an Agilent 7890 gas chromatograph coupled with a time-of-flight mass spectrometer. A 1 μL sample was injected with splitless mode to a DB-5MS capillary column (30 m × 250 μm × 0.25 μm, J&W Scientific, Folsom, CA, USA) with helium as the carrier gas at a flow of 1 mL min^−1^. The initial temperature of the column was kept at 50 °C for 1 min, then raised to 310 °C at a rate of 10 °C min^−1^, and then finally held for 8 min at 310 °C. The injection, transfer line, and ion source temperatures were 280, 280 and 250 °C, respectively. The energy was − 70 eV in electron impact mode. The mass spectrometry data were acquired in full-scan mode with the m/z range of 50–500 at a rate of 12.5 spectra per second after a solvent delay of 6.35 min. Raw data analysis, including peak extraction, baseline adjustment, deconvolution, alignment and integration, was finished with Chroma TOF (V 4.3x, LECO) software. LECO-Fiehn Rtx5 database was used for metabolite identification by matching the mass spectrum and retention index. Moreover, the peaks detected in less than half of QC samples or RSD > 30% in QC samples were removed.

### Targeted metabolomics

To assess changes in central carbon metabolism with altitudinal gradients, including glycolysis, pentose phosphate pathway (PPP), and tricarboxylic acid cycle (TCA cycle), targeted metabolomics was conducted to determine the content of 52 metabolites using HPIC-MS/MS (AB Sciex QTRAP® 6500 + , USA). Six samples per group were selected for high-throughput targeted metabolomics analysis. Liver tissues were precisely weighed and spiked with 500 μL pre-cooled methanol: H_2_O (3:1, v:v). The mixture was homogenized for 4 min at 40 Hz and sonicated for 5 min in an ice-water bath. Then samples were incubated at − 40 °C for 1 h and centrifuged at 13,800 g, 4 °C for 15 min. The supernatants (400 μL) were collected and dried by spin. Dried residue was dissolved in 250 µL of water. Reconstituted solution was vortexed before filtration through the filter membrane, and subsequently transferred to injection vials for HPIC-MS/MS analysis.

The HPIC separation was carried out using a Thermo Scientific Dionex ICS-6000 HPIC System (Thermo Scientific), equipped with Dionex IonPac AS11-HC (2 mm × 250 mm) and AG11-HC (2 mm × 50 mm) columns, as well as coupled to an AB SCIEX 6500 QTRAP + triple quadrupole mass spectrometer (AB Sciex) in the multiple reaction monitoring (MRM) mode. The mobile phase A was 100 mM NaOH in water, and the mobile phase D was ultrapure water. The column temperature was set at 30 °C. The auto-sampler temperature was set at 4 °C and the injection volume was 5 μL. Typical ion source parameters were set as follows: ion spray voltage = − 4500 V, temperature = 450 °C, ion source gas 1 = 45 psi, ion source gas 2 = 45 psi, curtain gas = 30 psi. AB SCIEX Analyst Work Station Software (1.6.3 AB SCIEX), MultiQuant 3.0.3 software and Chromeleon7 were employed for MRM data acquisition and processing.

### Enzyme activity

The activity of enzymes in liver of *B. gargarizans* was assayed using commercial kits from two companies: Beijing Solarbio Science & Technology Ltd. Co., Beijing, China; and Nanjing Jianjian Co., Ltd., Nanjing, China. All assays were performed at 25 ± 0.5 °C using a UV–visible spectrophotometer (UV-5100H, METASH, Shanghai, China). PDH catalyzes the dehydrogenation of pyruvate with a reduction of 2,6-dichlorophenophenol (2,6-DCPIP) at 605 nm. One unit of PDH activity was defined as the consumption of 1 nmol of 2, 6-DCPIP per milligram of tissue protein per minute (U mg^−1^ protein). Isocitrate and nicotinamide adenine dinucleotide (NAD^+^) (oxidized form) can be catalyzed by ICDH to produce* α*-ketoglutarate and nicotinamide adenine dinucleotide (reduced form) (NADH). One unit of ICDH activity was defined as production of 1 nmol of *α*-ketoglutarate per milligram of tissue protein per minute (U mg^−1^ protein). The reverse reaction of the process in which LDH catalyzes the formation of lactate from pyruvate. LDH catalyzes the reverse of the reaction in which pyruvate generates lactate. Then, pyruvate reacts with 2,4-dinitrophenylhydrazine to form pyruvate dinitrophenylhydrazone, that is brownish-red in alkaline solution. One unit of LDH activity was defined as the production of 1 μmol pyruvate per milligram of tissue protein for 15 min (U mg^−1^ protein) at 440 nm. SDH catalyzes the dehydrogenation of succinate to form fumarate, and the removed hydrogen is transferred through phenazine methosulfate (PMS) to reduce 2,6-DCPIP. One unit of SDH activity was defined as a decrease of 0.01 in absorbance of the reaction system per milligram of tissue protein per minute (U mg^−1^ protein) at 600 nm. HK and G6PDH catalyze reactions accompanied by nicotinamide adenine dinucleotide phosphate (NADPH) production. Thus, one unit of both HK and G6PDH activity was defined as the production of 1 nmol NADPH per milligram of tissue protein per minute (U mg^−1^ protein) at 340 nm. *α*-KGDH converts NAD^+^ to NADH in the process of catalyzing the formation of succinyl-CoA. One unit of *α*-KGDH activity was defined as the production of 1 nmol NADH per milligram of tissue protein per minute (U mg^−1^ protein). GDH, PK, and PFK can all oxidize NADH to NAD^+^, and thus their activities were reflected by measuring the decrease of NADH at 340 nm. One unit of activity for these three enzymes was defined as 1 nmol of NADH consumed per milligram of tissue protein per minute (U mg^−1^ protein). In addition, protein concentration was assayed by the Bradford dye-binding method using bovine serum albumin as the standard [32].

### Statistical analysis

Non-targeted and targeted metabolomics data were tested for the normality of distribution and homogeneity of variance using Shapiro–Wilk and Levene’s tests. If the assumptions were met, data were assessed using a one-way ANOVA. Data that did not meet the assumptions were evaluated using Welch’s t test. All metabolites from the target metabolomics and non-target metabolomics were analyzed separately by k-means clustering, and then those metabolites in each cluster were subjected to Kyoto Encyclopedia of Genes and Genomes (KEGG) enrichment analysis. Moreover, a weighted gene co-expression network analysis (WGCNA) was performed for all metabolites in the non-target metabolomics to identify modules associated with altitude. The key parameters of WGCNA include softPower = 27, minModuleSize = 10, and mergeCutHeight = 0.25 × 10^–5^. Pearson's correlation coefficient was also calculated to determine the correlation of each module with altitude. After that, the top modules significantly correlated with altitude were selected for KEGG enrichment analysis. All KEGG enrichment analyses were performed in MetaboAnalyst 5.0 (http://www.metaboanalyst.ca). *P* < 0.05 indicates significant enrichment of pathways.

The data for enzyme activity were analyzed using SPSS 22.0 software (SPSS, Inc., Chicago, Illinois, USA). A general linear model (GLM) followed by Bonferroni-corrected pairwise comparisons were used to investigate the effect of altitude on the activity of enzymes, with altitude as a fixed factor and body mass as a covariate. A significance level of *P* < 0.05 was accepted in all cases.

## Results

### Non-targeted metabolomics

**Fig. 1:**
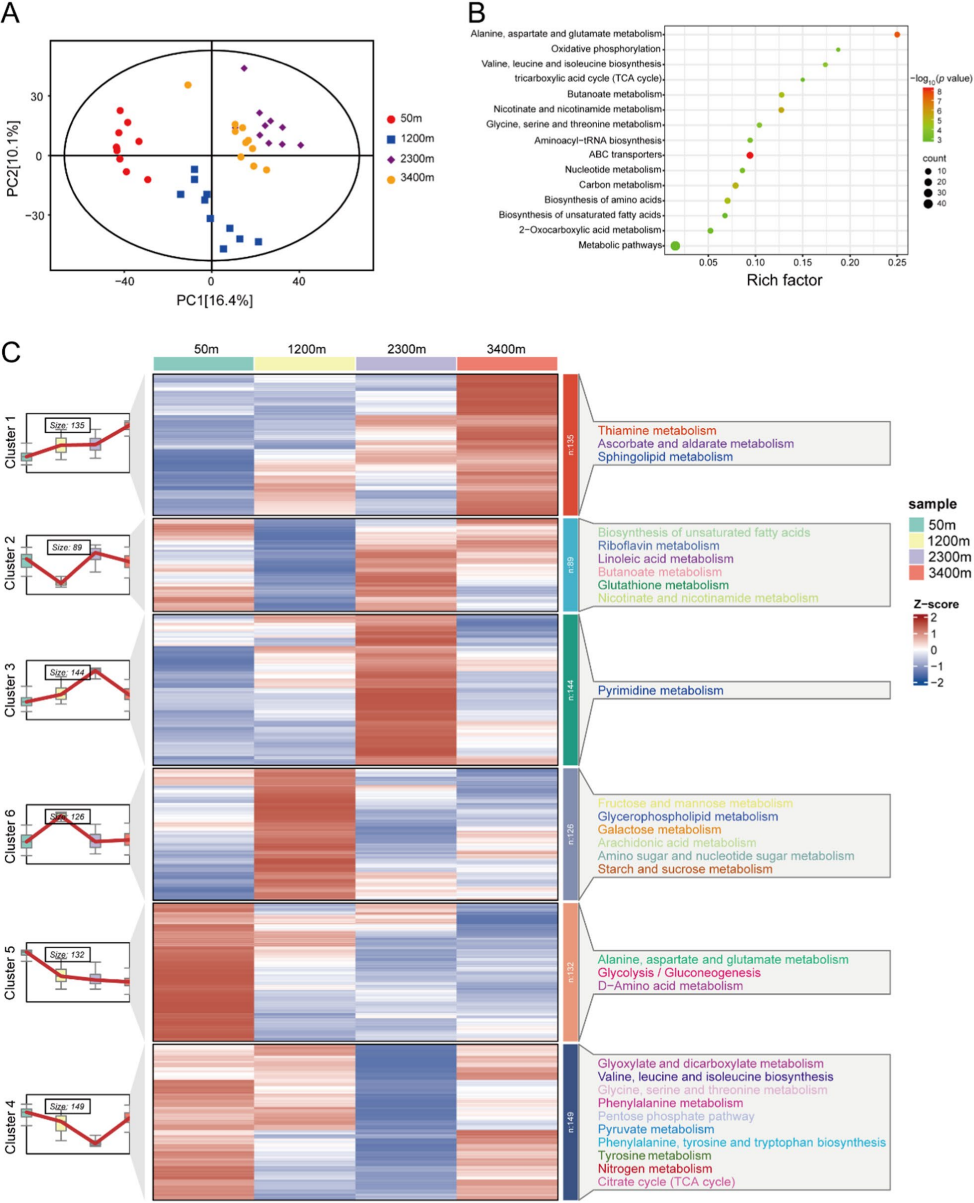
**A** Principal component analysis (PCA) score plot. Four groups are distinguished: 50 m altitude, red circles; 1200 m altitude, blue boxes; 2300 m altitude, purple diamonds; and 3400 m, yellow circles. **B** KEGG pathway enrichment analysis for differential metabolites in liver of *B. gargarizans*. Color shades and circle sizes represent log_10_(*P* value) and metabolite numbers, respectively. **C** Heat map of the expression pattern of differential metabolites in *B. gargarizans* at different altitudes. Red and blue colors indicate the up-regulation and down-regulation, respectively

Using UHPLC-QE-MS and GC-TOF–MS, a total of 15,569 valid peaks were extracted and 775 metabolites were identified using an in-house standard and public database. The PCA score plot showed that PC1 and PC2 explained 16.4% and 10.1% of the features of the original dataset, respectively, and that the four groups of samples displayed a clear separation (Fig. 1A). A total of 520 significantly different metabolites (SDMs) were screened by a condition of *P* < 0.05. KEGG pathway enrichment analysis showed that these SDMs were mainly enriched in metabolic pathways, including nucleotide metabolism, oxidative phosphorylation, 2-oxocarboxylic acid metabolism, TCA cycle, and biosynthesis of unsaturated fatty acids (Fig. 1B). K-means clustering analysis showed that all metabolites were classified into six clusters. As altitude rose from 50 to 3400 m, a total of 135 metabolites in cluster 1 showed remarkable increases, whereas 132 metabolites in cluster 5 decreased. KEGG pathway analysis showed that metabolites in cluster 1 were mainly enriched in thiamine metabolism, ascorbate and aldarate metabolism, and sphingolipid metabolism (Fig. 1C). Metabolites in cluster 5 were mainly enriched in alanine, aspartate and glutamate metabolism, glycolysis/glycogenesis, and d-amino acid metabolism (Fig. 1C). Metabolites in other clusters were enriched in biosynthesis of unsaturated fatty acids, riboflavin metabolism, glyoxylate and dicarboxylate metabolism, valine, leucine and isoleucine biosynthesis, fructose and mannose metabolism, glycerophospholipid metabolism, and pyrimidine metabolism (Fig. 1C).

### WGCNA and key module identification

**Fig. 2:**
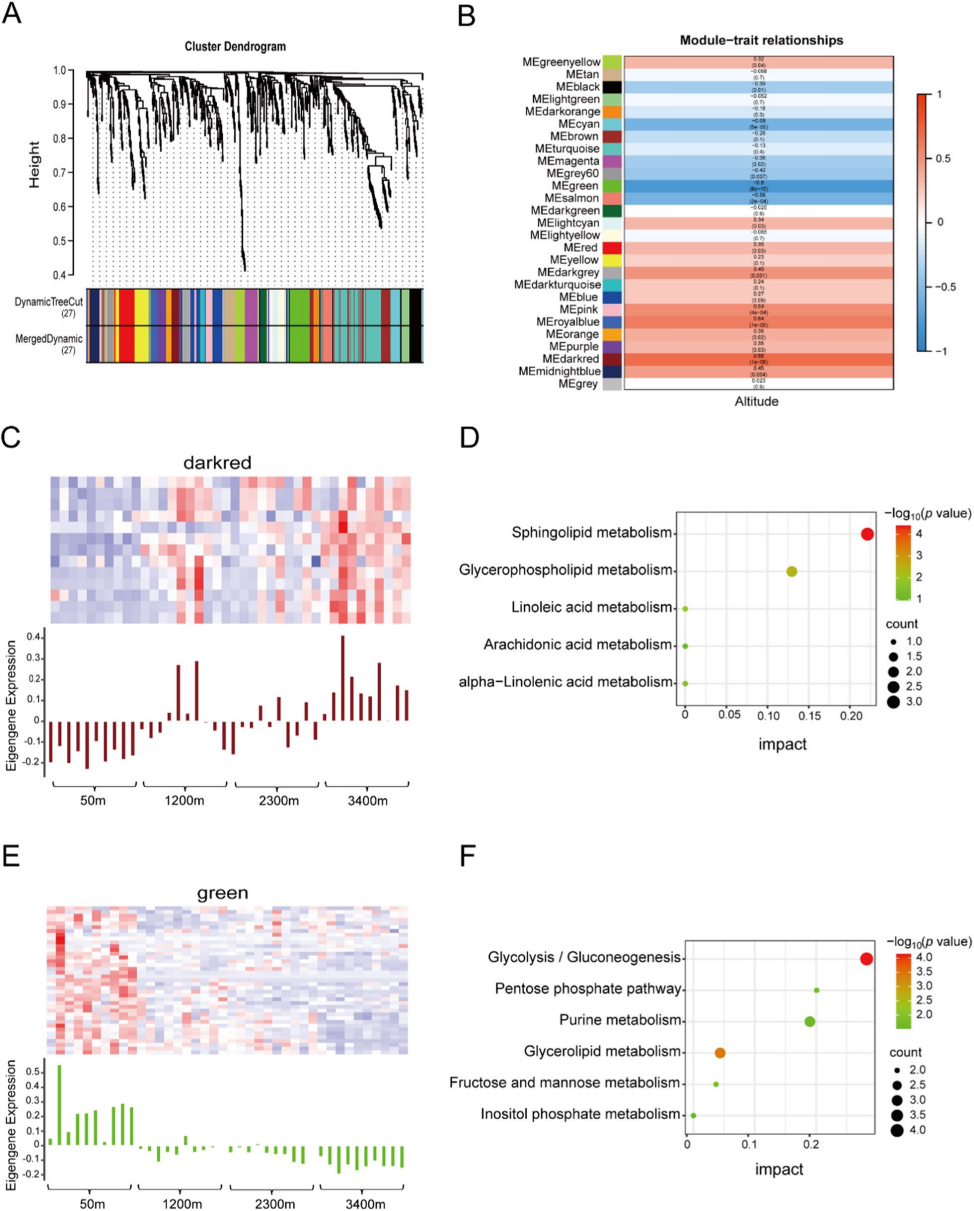
**A** Clustering dendrograms of metabolites were obtained by Topological Overlap Matrix (TOM). Below the dendrogram, the first row represents the assigned original modules while the second row displays the merged modules. **B** Correlation of modules with altitude was analyzed using WGCNA. Rows indicate 27 metabolite modules and columns indicate elevation. The modules at the top correlated significantly with altitude, including dark red and green. Co-expression heat maps of metabolites within the **C** darkred and **E** green modules (upper panel) and expression of corresponding metabolites in each sample (lower panel). Differential metabolites enrichment of KEGG pathways in **D** darkred and **F** green modules. The color shades represent log_10_(*P* value), and the size of the circle represents the metabolite numbers

A total of 27 different co-representation modules were constructed (Fig. 2A), in which 16 modules were significantly correlated with altitude (*P* < 0.05) (Fig. 2B), and darkred module showed the highest positive correlation (r = 0.68) with altitude (Fig. 2C). However, the green module showed the highest negative correlation (r = − 0.08) with altitude (Fig. 2E). Metabolites in the darkred module were significantly enriched in pathways such as sphingolipid metabolism, glycerophospholipid metabolism, linoleic acid metabolism, and* α*-linolenic acid metabolism (Fig. 2D). Metabolites in the green module were significantly enriched in glycolysis/gluconeogenesis, glycerolipid metabolism, fructose and mannose metabolism, PPP, purine metabolism, and inositol phosphate metabolism (Fig. 2F).

### Targeted metabolomics

**Fig. 3:**
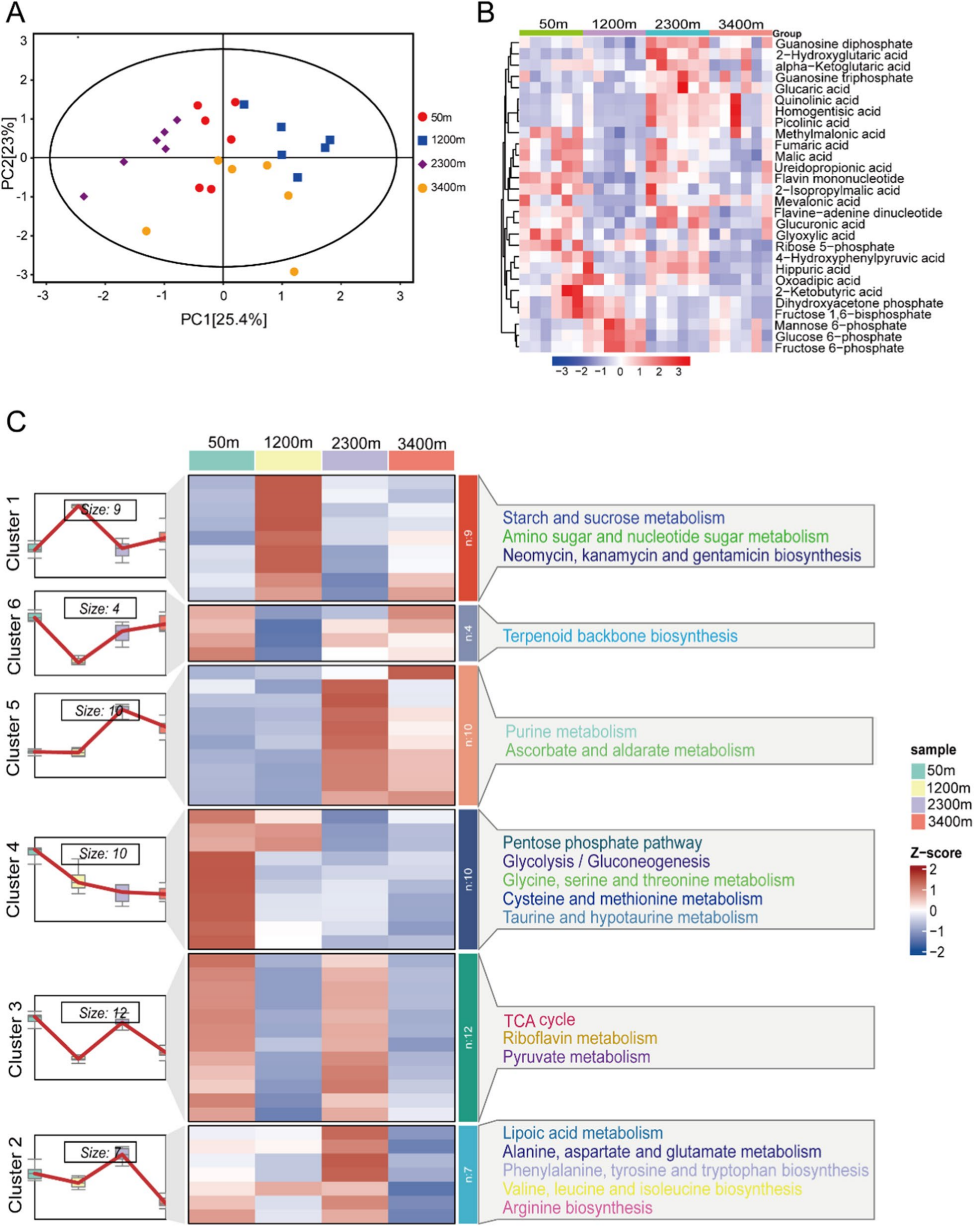
**A** Principal component analysis (PCA) score plot. Four groups were distinguished: 50 m altitude, red circles; 1200 m altitude, blue boxes; 2300 m altitude, purple diamonds; and 3400 m, yellow circles. **B** Heat map showing the relationships and changes in the relative abundance of SDMs among the four altitudes. Red color indicates high-abundance metabolites, and blue indicates low-abundance metabolites. **C** Heat map of the expression pattern of differential metabolites in *B. gargarizans* at different altitudes. Red and blue colors indicate the up-regulation and down-regulation, respectively

Although the PCA showed a tendency of group separation, the overlap among samples indicates moderate group-level differences in metabolite composition. PC1 and PC2 together explained 48.4% of the variance (Fig. 3A). A total of 28 SDMs were screened (*P* < 0.05) (Fig. 3B). K-means clustering analysis showed that all metabolites were classified into six clusters. A total of 10 metabolites in cluster 4 decreased with increasing altitude, which was mainly enriched in PPP, glycolysis/gluconeogenesis, glycine, serine and threonine metabolism, cysteine and methionine metabolism, and taurine and hypotaurine metabolism (Fig. 3C). Moreover, a total of 10 metabolites in cluster 5 increased with rising altitude but decreased at 3400 m. These metabolites were mainly enriched in purine metabolism, and ascorbate and aldarate metabolism (Fig. 3C). Besides these two clusters, metabolites in the other clusters were mainly enriched in starch and sucrose metabolism, amino sugar and nucleotide sugar metabolism, lipoic acid metabolism, alanine, aspartate and glutamate metabolism, phenylalanine, tyrosine and tryptophan biosynthesis, TCA cycle, riboflavin metabolism, and terpenoid backbone biosynthesis (Fig. 3C).

### Enzyme activity

**Fig. 4:**
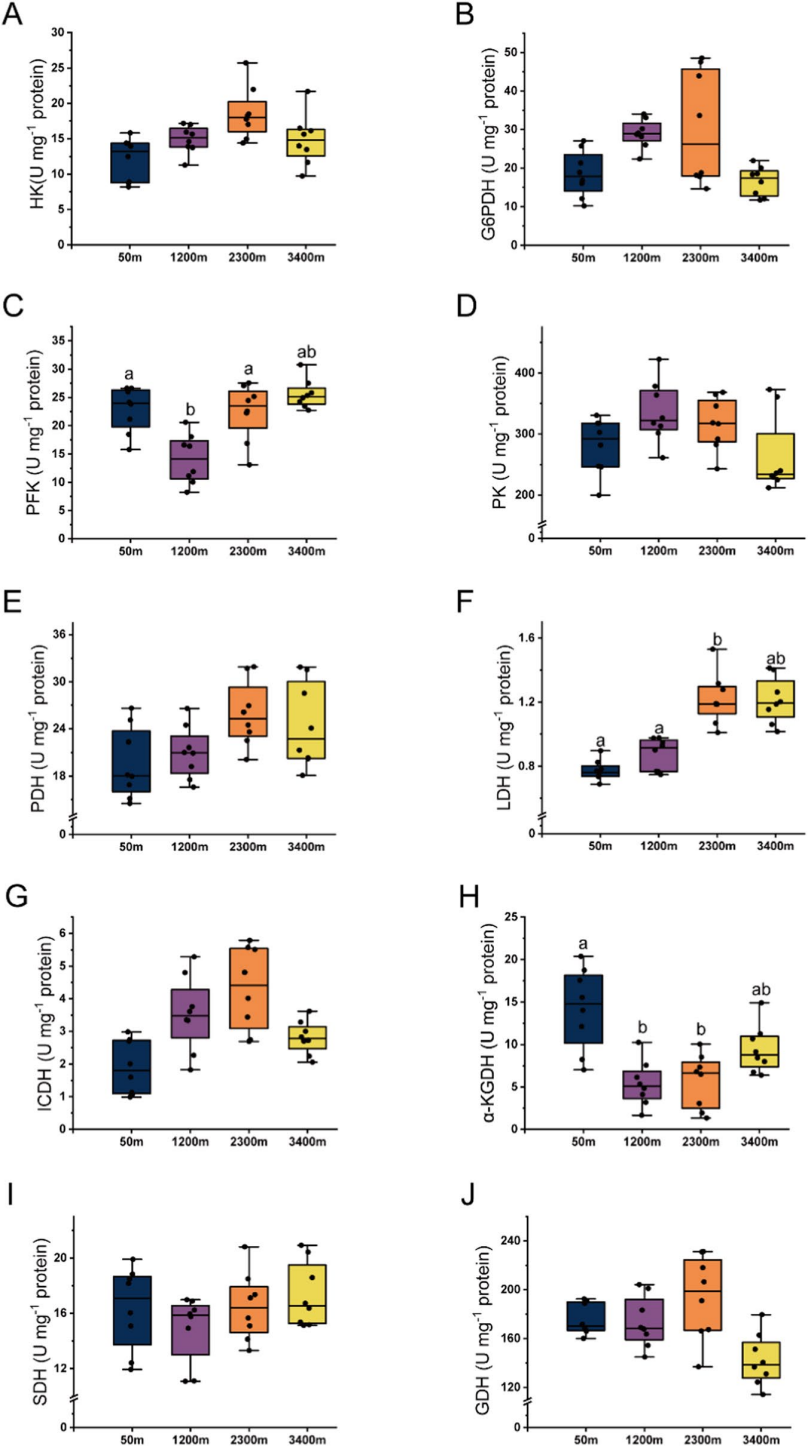
Enzyme activity in the liver of *B. gargarizans* from different altitudes, including **A** hexokinase (HK), **B** 6-phosphoglucokinase (G6PDH), **C** phosphofructokinase (PFK), **D** pyruvate kinase (PK), **E** pyruvate dehydrogenase (PDH), **F** lactate dehydrogenase (LDH), **G** isocitrate dehydrogenase (ICDH), **H** *α*-ketoglutarate dehydrogenase (*α*-KGDH), **I** succinate dehydrogenase (SDH), and **J** glutamate dehydrogenase (GDH). Different letters indicate significant differences between different altitudes (*P* < 0.05)

Metabolic enzyme activities did not differ significantly among different altitudes, including HK (Fig. 4A), G6PDH (Fig. 4B), PK (Fig. 4D), PDH (Fig. 4E), ICDH (Fig. 4G), SDH (Fig. 4I), and GDH (Fig. 4J). However, the activity of PFK (Fig. 4C), LDH (Fig. 4F), and* α*-KGDH (Fig. 4H) showed significant changes along the altitudinal gradient (*P* < 0.05). PFK activity was lowest at 1200 m altitude, but there was no significant difference between the altitudes of 1200 m and 3400 m. LDH activity was significantly higher at 2300 m altitude than that at altitudes of 50 m and 1200 m. However, LDH activity at 3400 m did not differ significantly compared to the values at other altitudes. The* α*-KGDH activity was highest at 50 m altitude, but no significant difference was observed between the altitudes of 50 m and 3400 m.

### Integrative analysis of metabolite and metabolic enzymes

**Fig. 5:**
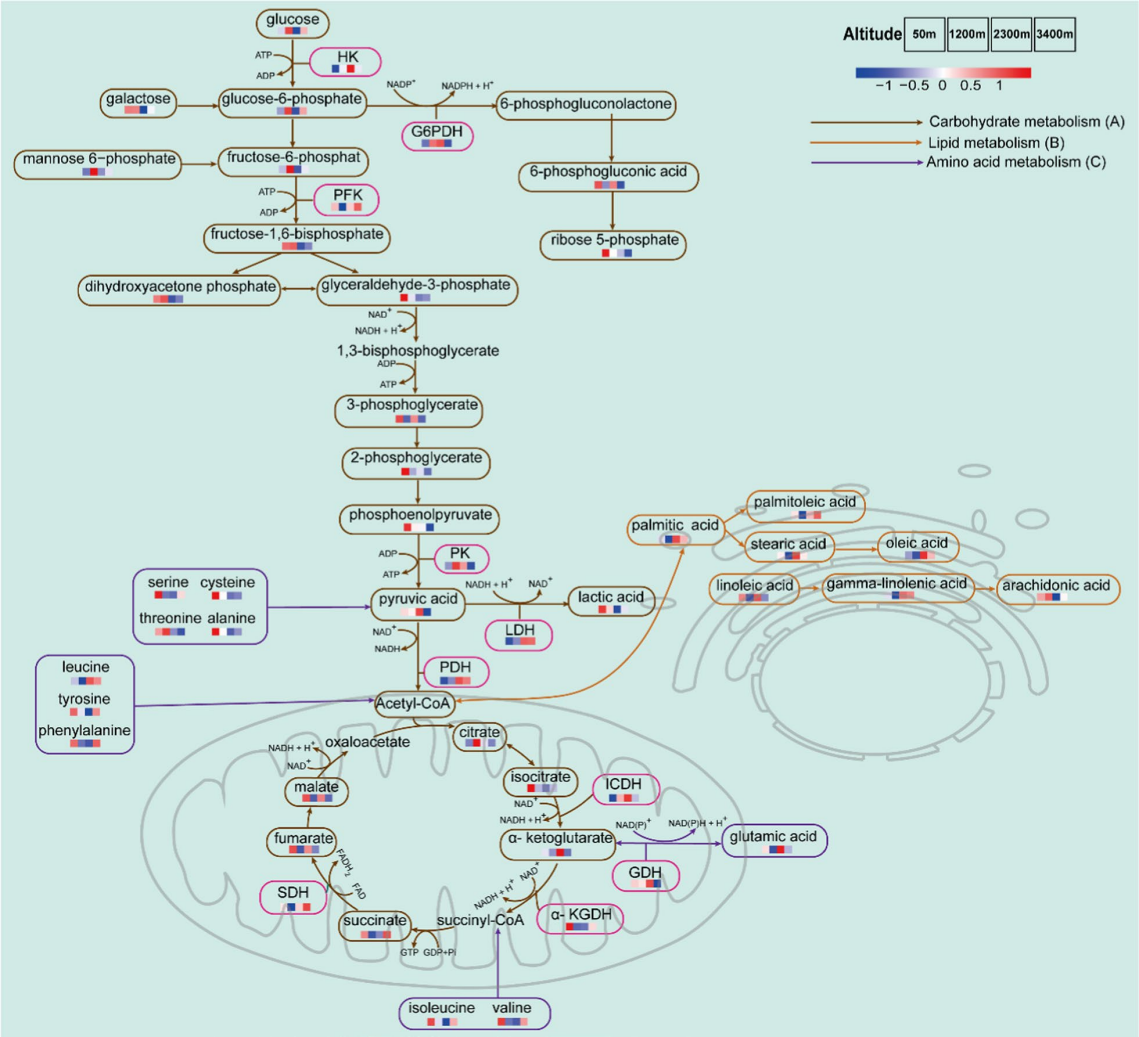
Primary changes in metabolites related to energy metabolism in the liver of *B. gargarizans* among four altitude gradients: **A** carbohydrate metabolism, **B** lipid metabolism, and **C** amino acid metabolism. Red color indicates the up-regulation, and blue indicates the down-regulation

Changes in metabolites and metabolic enzymes reflected the major energy metabolism pathways including carbohydrate metabolism, amino acid metabolism, and fatty acid metabolism (Fig. 5). Five intermediates of glycolysis showed a decreasing trend with rising altitude, including 3-phosphoglyceraldehyde, 3-phosphoglycerate, 2-phosphoglycerate, phosphoenolpyruvate, and dihydroxyacetone phosphate. Along the altitudinal gradient, the contents of palmitoleic, oleic, linolenic, and palmitic acids tended to increase, but some amino acids tended to decrease, such as aspartate, threonine, alanine, and valine. LDH and ICDH activity tended to increase along the altitude gradient, whereas* α*-KGDH activity tended to decrease.

## Discussion

We explored the hepatic metabolic profiles of *B. gargarizans* along an altitudinal gradient using multi-platform non-targeted (including UHPLC-QE-MS and GC-TOF–MS) and targeted (HPIC-MS/MS) metabolomics approaches. The present findings showed changes in carbohydrate metabolism, amino acid metabolism, purine metabolism, and lipid metabolism, and provided new insights into the adaptation of amphibians to environmental conditions at different altitudes.

### Carbohydrate metabolism

Hypoxia-tolerant ectothermic vertebrates typically enter an energy-saving hypometabolic state by strongly inhibiting energy production and consumption to cope with oxygen deficiency [33]. Thus, the down-regulation of glycolysis and non-essential pathways like PPP in high-altitude toads contributes to metabolic rate depression (MRD), that can effectively decelerate the consumption of fuel/energy reserves. MRD has been widely demonstrated in many animal species dwelling at high altitudes [14, 34]. The PPP was depressed with increasing altitude, consistent with previous findings that metabolic fluxes of the PPP decreased significantly along the altitudinal gradient [29]. It has been demonstrated that both hypoxia and low-temperature stresses lead to significant inhibition of glycolysis and PPP in the liver of ectothermic vertebrates [20, 35, 36]. Indeed, mean annual temperature and oxygen partial pressure in the four sampled cities decreased with increasing altitude. These variations may contribute to the observed down-regulation of metabolites in the glycolysis and PPP. A previous study showed that the expression of *LDHD* was lower at 2300 m than at 50 m and 1200 m altitudes [29]. However, we found that LDH activity at 2300 m was significantly higher than that at 50 m and 1200 m. This may be attributed to post-translational modifications, which can critically influence LDH activity. Indeed, acetylation has been shown to affect LDH activity in the frog, *Rana sylvatica* and the turtle, *Trachemys scripta elegans* [37, 38]. Similar to *P. vlangalii* and *P. erythrurus* living at high altitude [15, 39], we also found no significant difference in the content of lactate in the toads among the four altitudes, which suggests that high-altitude toads can prevent acidosis caused by lactate accumulation.

### Amino acid metabolism

Hypoxia exposure resulted in an increased level of alanine in *Rana amurensis* [40]. However, seasonal cold leads to a significant reduction in alanine content in the lizard, *Lacerta vivipara* [41]. Thus, the low temperature at high altitude was a potential contributor to the decrease in alanine. Moreover, alanine is primarily synthesized through transamination reactions, with pyruvate serving as its key substrate [42]. A lower level of alanine was associated with depressed glycolysis/gluconeogenesis in high-altitude toads. Similar results were observed in the lizard, *P. vlangalii*, where the level of alanine was reduced in the liver at high altitudes [15]. Glycine, glutamic acid, and cysteine can be used to synthesize glutathione [43], that may be responsible for the lower level of cysteine and glycine in high-altitude individuals. Indeed, we found a higher level of glutathione in the liver of high-altitude toads. Glutathione can directly scavenge free radicals and peroxides, thereby protecting cells from oxidative damage [44]. The higher level of glutathione can contribute to enhancing antioxidant defenses to cope with high-altitude hypoxia rather than low-temperature stress, since previous findings suggests that chronic cold exposure leads to decreased glutathione in the liver of high-altitude frogs, *Nanorana pleskei* [45]. A previous study also demonstrated that *B. gargarizans* living at high altitudes possess strong antioxidant defenses [46].

### Purine metabolism

K-means clustering analysis showed that the level of guanosine triphosphate (GTP) was higher in the liver of high-altitude toads (2300 m and 3400 m) than those in low-altitude toads (50 m and 1200 m). It has been shown that GTP influences the structure of hemoglobin as a ligand and regulates its affinity for oxygen [47]. Cold exposure is generally associated with a reduction in intracellular GTP levels, because low temperatures suppress overall metabolic activity, including nucleotide biosynthesis and ATP/GTP turnover [48]. For instance, GTP content in the tissue of mussels (*Mytilus galloprovincialis*) was reduced during the cold season (winter) [49]. Thus, higher levels of GTP may regulate the oxygen affinity of hemoglobin that is likely beneficial for hypoxia survival at high altitudes. We also observed a decrease in the level of hypoxanthine with increasing altitude, but no significant difference was found in the content of xanthine among the four altitudes. Numerous studies have demonstrated that the accumulation of xanthine and hypoxanthine can lead to oxidative stress [31, 50]. Therefore, the lower level of hypoxanthine suggests that high-altitude environmental factors did not lead to oxidative stress in toads, which is consistent with previous findings [46].

### Lipid metabolism

It is well known that phosphatidylcholine (PC) and phosphatidylethanolamine (PE) are important components of cell membranes. A previous study showed that PCs and PEs play an important role in maintaining membrane fluidity and structural integrity [51]. Moreover, low temperatures lead to an increased ratio of PE/PC (or a decrease in PC/PE ratio) in ectotherms, which contributes to counteracting reduced membrane fluidity [52]. However, k-means clustering analysis showed that 10 PCs were positively correlated with the altitudinal gradient, whereas 3 PCs, 1 PI, and 2 PEs were all negatively correlated with the altitudinal gradient. This would lead to a higher ratio of PC/PE, that appears to be detrimental to maintaining membrane fluidity at low temperature. Similar results were observed in rainbow trout *Oncorhynchus mykiss*, where low-temperature (5 °C) acclimation resulted in elevated PC levels in raft regions of hepatocyte plasma membranes [53]. In addition to the PE/PC ratio, unsaturated fatty acids play a key role in increasing membrane fluidity and maintaining membrane structural stability [54]. Moreover, low temperature leads to an increase of unsaturated fatty acids in *N. parkeri* and *Rana esculenta* [31, 55]. In this study, the metabolites of cluster 2 that showed an increasing trend with rising altitude were significantly enriched in the biosynthesis of unsaturated fatty acids, which contributes to improving membrane fluidity and ensuring normal cell membrane function at low temperatures. Many studies have demonstrated that fatty acid metabolism was reduced in many animal species living in hypoxic environments, with a lower level of phospholipids and fatty acids [56, 57]. Thus, these changes in phospholipids and unsaturated fatty acids in toads were largely driven by low temperatures rather than low oxygen levels at high altitude. Cholesterol plays an important role in protecting membrane lipids from oxidative damage and regulating the fluidity of the membrane [58, 59]. We found that the content of cholesterol increased with rising altitude, which may be an adaptation by the toads to the high plateau environment.

## Conclusions

In summary, this is the first study to explore the metabolic profiles of liver in the Asiatic toad (*B. gargarizans*) along an altitudinal gradient. Toads appear to adopt an MRD strategy to survive at high altitudes by down-regulating glycolysis, the PPP, and glycerolipid metabolism. As altitude rises, toads enhance their protective mechanisms, such as increased levels of glutathione and GTP, to cope with hypoxic stress at high altitudes. The up-regulation of the biosynthesis of unsaturated fatty acids and the higher level of cholesterol may contribute to maintaining membrane fluidity and protecting membrane integrity, which is crucial for survival of low temperatures at high altitudes. These findings reveal the adaptation of toads to high-altitude environments and provide insights to better understand the survival strategy of ectotherms in extreme environments. Since populations at different altitudes and latitudes are separated by both geographic distance and genetic background, observed metabolic differences may reflect both environmental and genetic divergence. Future transplant studies are needed to confirm the causative role of altitude.

## Data Availability

The datasets used and/or analysed during the current study are available from the corresponding author on reasonable request.
